# Traditional South African medicinal plants and their role in improving goat reproduction: a review

**DOI:** 10.3389/fvets.2026.1729085

**Published:** 2026-04-13

**Authors:** Kamogelo Shai, Nthabiseng Amenda Sebola

**Affiliations:** Department of Agriculture and Animal Health, College of Agriculture and Environmental Sciences, University of South Africa, Johannesburg, South Africa

**Keywords:** *Aloe ferox* mill, ethnoveterinary, *Moringa oleiffera* lam, pharmacology, *Securidaca longepedunculata* Fresen

## Abstract

**Introduction:**

Goats are an integral part of the livelihoods of South Africans, particularly in the rural communities, yet reproductive inefficiencies (low libido, poor semen quality, uterine infections, postpartum disorders, parasitism, and nutrition gaps) limit their productivity. Most resource challenged farmers resort to the use of ethnoveterinary plants for reproductive health of their goats due to their safety and ease of access; however, there is still a lack of standardization on their safety, dosing and efficacy.

**Aim:**

This study aimed to review and document the South African traditional medicinal plants used to enhance reproductive performance in goats.

**Methods:**

The study carried out a narrative review of ethnoveterinary surveys and pharmaco-ethnobotanical literature focused on South Africa, complemented by relevant goat reproduction studies. Our search used various keywords, including “medicinal plants,” “goat breeding,” “ethnoveterinary,” and “medicinal plants” to identify relevant literature in several databases, including Scopus, Web of Science, Access to Global Online Research in Agriculture, and ScienceDirect. Additional searches were conducted using citations found in articles in these databases. The focus was on peer reviewed journals published between the year 2000 and 2025 on South African medicinal plants used to enhance goat reproduction, whether directly or indirectly.

**Overview of key insights:**

During the literature review, it was found that among other plants *Securidaca longepedunculata* Fresen. (violet tree), *Moringa oleifera* Lam (moringa), *Elephantorrhiza elephantina* (Burch.) Skeels (elephant root), *Kigelia africana* (Lam.) Benth. (sausage tree), *Aloe ferox* Mill., were frequently mentioned. Strong evidence was noted from ethnobotanical use to *in vitro/in vivo* validation, though limited but growing, especially for anthelmintic and antioxidant actions.

**Conclusion:**

Based on the literature, it can be concluded that South Africa’s ethnobotanical resources hold credible value for improving goat reproduction by acting as antioxidants, regulating hormones, fighting infections, and controlling parasites that affect body condition. However, well-designed goat studies with proper dosing and safety testing are limited.

## 1 Introduction

In rural communities of South Africa, goats play a crucial role by contributing substantially to food security and cash income (1–3, 51). Reproductive efficiency is driven by libido and semen quality in bucks, and uterine health, postpartum recovery in does; furthermore, body condition largely determines off-take and profitability. While conventional therapeutics and mineral supplementation underpin modern herd health, cost and access barriers keep many smallholder farmers reliant on ethnoveterinary medicine, due to their affordability and accessibility, especially in Eastern Cape, Limpopo, and Northwest provinces (2, 4–6). In addition, the upsetting increase in drug resistant and chemical residues in animal products worldwide surges the need to opt for alternative, safer possibilities such as medicinal plants (7, 8) since there is a universal belief that products from nature are harmless and more harmonious with biological systems (9). Livestock farmers in South Africa have used medicinal plants for centuries to manage various livestock disorders (10). This ethnoveterinary practice is an important part of their culture, a practice that is likely to carry over from generation to generation (11). Masika et al. (12) reported that about 75% of resource challenged farmers in the Eastern Cape Province rely fully on medicinal plants to treat their livestock and it is believed that these plant remedies carry pharmacologically active compounds (13) However, it has been noted that there is a lack of repositories on ethnoveterinary information relating to goat reproduction as it is passed orally from older generations to the young ones (2). Therefore, there is a risk that some knowledge may be lost during the transfer, resulting in an indigenous knowledge gap. There is a pool of documented literature on the use of plant medicine in goats; however, the focus is mainly on parasite control rather than direct reproductive endpoints, highlighting a knowledge gap in reproductive performance oriented ethnoveterinary research (14–16) Our review aims to (i) Collate South African traditional plants reported for improving reproductive performance in goats, (ii) summarize preparation/uses, and (iii) sketches plausible mechanisms.

## 2 Materials and methods

**Figure 1:**
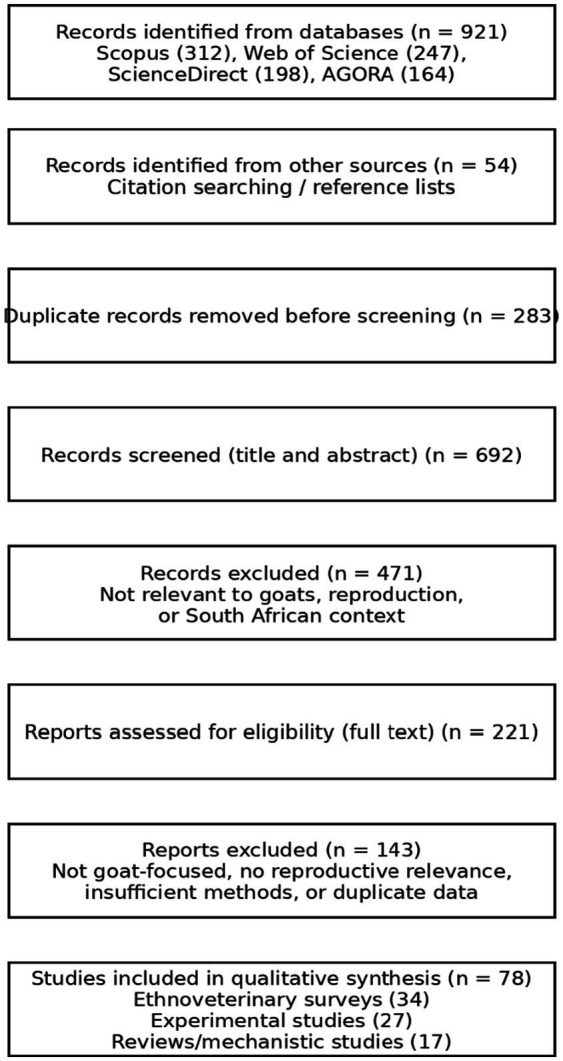
PRISMA 2020 flow diagram illustrating the identification, screening, eligibility assessment, and inclusion of studies on South African medicinal plants relevant to goat reproductive health (2000–2025). Studies were synthesized qualitatively; no meta-analysis was performed.

The data for this review come from various databases in which peer-reviewed journals, books and conference proceedings have been published. Various keywords such as “medicinal plants, goat farming, ethnoveterinary” and medicinal plants were used alone or in combination to identify appropriate literature from multiple databases. Careful selection of these keywords led the author to consult databases such as Scopus, Web of Science, Access to Global Online Research in Agriculture and Science Direct. In addition, searches were carried out using citations found in articles in the database. The focus was on peer reviewed journals published between the year 2000 and 2025 on South African medicinal plants used to enhance goat reproduction, whether directly or indirectly. The literature selection process followed the PRISMA 2020 guidelines for transparent reporting of evidence identification and screening. Although this study was not designed as a systematic review or meta-analysis, a PRISMA 2020 flow diagram was used to document the identification, screening, eligibility, and inclusion of relevant studies. Due to the qualitative, heterogeneous, and ethnobotanical nature of the available literature, the included studies were synthesized narratively rather than quantitatively (Figure 1).

## 3 Literature review

### 3.1 Ethnoveterinary knowledge in South Africa

Ethnoveterinary knowledge is widely defined as the use of cultural practices, beliefs, and indigenous plants to maintain overall animal health and productivity (17). Ethnoveterinary in most provinces of South Africa is deeply rooted in communities where farming is an integral part of their lives and this knowledge is mediated by farmers, traditional healers, and herbalists and is passed down through generations (15). Medicinal plants are an alternative source to conventional veterinary services and pharmaceuticals, as these are beyond the reach of the majority of smallholder and communal farmers due to financial constraints or unavailability (18). Research has been carried out in various provinces of South Africa, such as Limpopo, Eastern Cape, KwaZulu-Natal, and Northwest where a wide range of medicinal plants are used in the management of livestock ailments, including reproductive problems (19, 48, 52), however, indications are without goat specific outcomes. Notably, Eastern Cape surveys categorized the use of medicinal plants against intestinal nematodes in goats as an indirect way of improving fertility through improved body condition (19). Farmers typically prepare remedies as decoctions, infusions, or powders, using leaves, roots, bark, or fruits depending on availability (19, 58). These remedies are applied to improve male fertility, assist with parturition, treat uterine infections, and manage parasitic burdens that indirectly influence reproductive performance (19, 20, 58). Despite its widespread use, ethnoveterinary knowledge faces challenges due to a lack standardized dosages in most practices and scientific validation, leading to variability in effectiveness and potential toxicity (21, 22). Furthermore, younger generations often show less interest in preserving this knowledge, putting it at risk of being lost (23), Nonetheless, documenting and scientifically testing ethnoveterinary practices in South Africa offers an important opportunity to improve goat reproduction in a culturally relevant and sustainable way.

### 3.2 Reproductive constraints in south African goats

Several interrelating factors challenge goat reproductive efficiency in South Africa, including, those that reduce fertility, conception rates, and kid survival. Poor nutrition, heat stress and parasitic burdens are among the factors that have been linked to poor goat performance wherein in bucks they result in low libido, reduced semen quality, and small scrotal size (24). In does these factors result in uterine infections, retained placenta, weak estrus expression, and early embryonic loss (25). Parasites such as *Haemonchus contortus* and tick-borne diseases indirectly impair reproduction by reducing body condition and blood parameters (53, 59). Seasonal feed shortages and trace mineral deficiencies [(Zinc (Zn), Selenium (Se), Copper (Cu), Phosphorus (P), Vitamin A, Vitamin E)] further compromise ovarian activity, spermatogenesis, and embryo viability (26, 27). In addition, lack of resources and knowledge or skills among most communal farmers result in extreme environmental and management problems such as heat stress, uncontrolled mating, poor record-keeping, and limited veterinary access thus result into reproductive inefficiencies. Collectively, these constraints highlight that improving reproductive performance requires an integrated approach that combines nutrition, parasite control, disease management, and buck evaluation, while also considering genetic influences between exotic and indigenous breeds and the effects of photoperiod on breeding activity. Cultural believes and lack of financial resources drive small holder farmers to rely on ethnoveterinary medicinal plants to address some of these challenges, particularly through their anthelmintic, antimicrobial, and antioxidant properties, however, scientific validation is still limited (Table 1).

**Table 1:** Summary of reproductive constraints in South African goats.

Constraints category	Key issues	Impact on reproduction	References
Bucks	Low libido, poor semen quality, small scrotal size	Reduced mating success, low sperm fertilization rates	(24)
Does	Metritis, retained placenta, weak estrus, embryo loss	Delayed conception, low kidding rates, higher mortality	(25)
Parasite and disease	Gastro intestinal nematodes (*Haemonchus contortus*), ticks, mastitis	Poor body condition, anemia, delayed cyclicity	(59)
Nutrition and minerals	Seasonal feed gaps, Zinc/Selenium/Copper/Phosphorus/Vitamins A/E deficiencies	Weak estrus, low sperm quality, embryo loss	(26, 27)
Environment and management	Heat stress, poor buck: doe ratios, weak records, no artificial insemination	Reduced libido, low conception, poor genetic progress	(27)
Socio economic	Limited veterinary services, feed costs, and low farmer knowledge	Continued reliance on traditional remedies	(12)

### 3.3 South African medicinal plants with reported reproductive use in goats

The literature search revealed a scant information on studies with a direct link to goat reproduction in South Africa. The information employed in this review was gathered from traditional ethnobotanical reports and goat studies done in other parts of Africa. The scarcity of information relating to goat reproduction urged the authors to use indirect possible relations, such as the use of medicinal plants to manage gastrointestinal parasites, which can improve body condition, which in turn may lead to better fertility.

#### 3.3.1 *Securidaca longepedunculata* Fresen.

**Figure:**
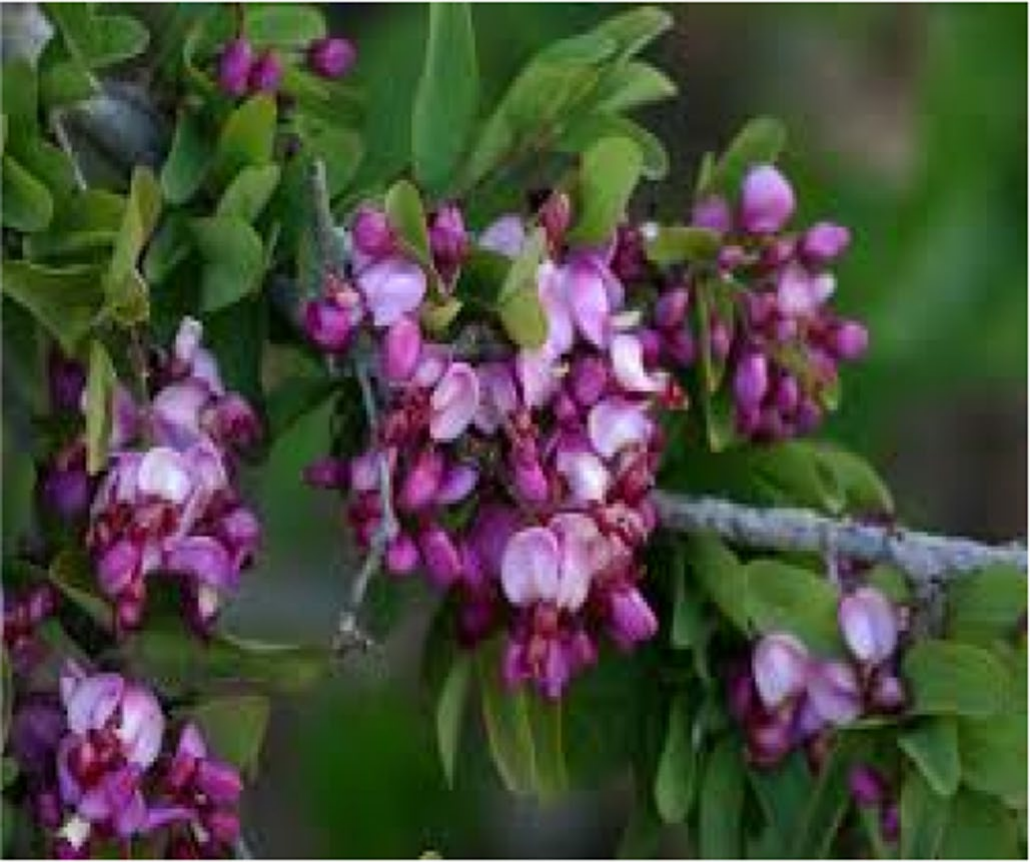

*S. longepedunculata* is traditionally used in South Africa as an aphrodisiac and libido stimulant, and it is regarded as a general reproductive enhancer for both men and livestock (20). The plant is rich in phytochemicals such as xanthones, saponins, and flavonoids, which have demonstrated antimicrobial and antioxidant properties. Recent South African studies have also profiled the chemical composition and biological activities of its plant parts, providing a scientific basis for its ethnoveterinary use (13). Mechanistically, the antioxidant and endocrine-modulating effects of these compounds may support spermatogenesis and libido, while the antimicrobial actions could help reduce subclinical infections that compromise reproductive health (20, 28). However, several studies have reported dose-dependent toxicity, particularly with root extracts, and a relatively low LD₅₀ in small mammals (29, 50).

#### 3.3.2 *Moringa oleifera* lam

**Figure:**
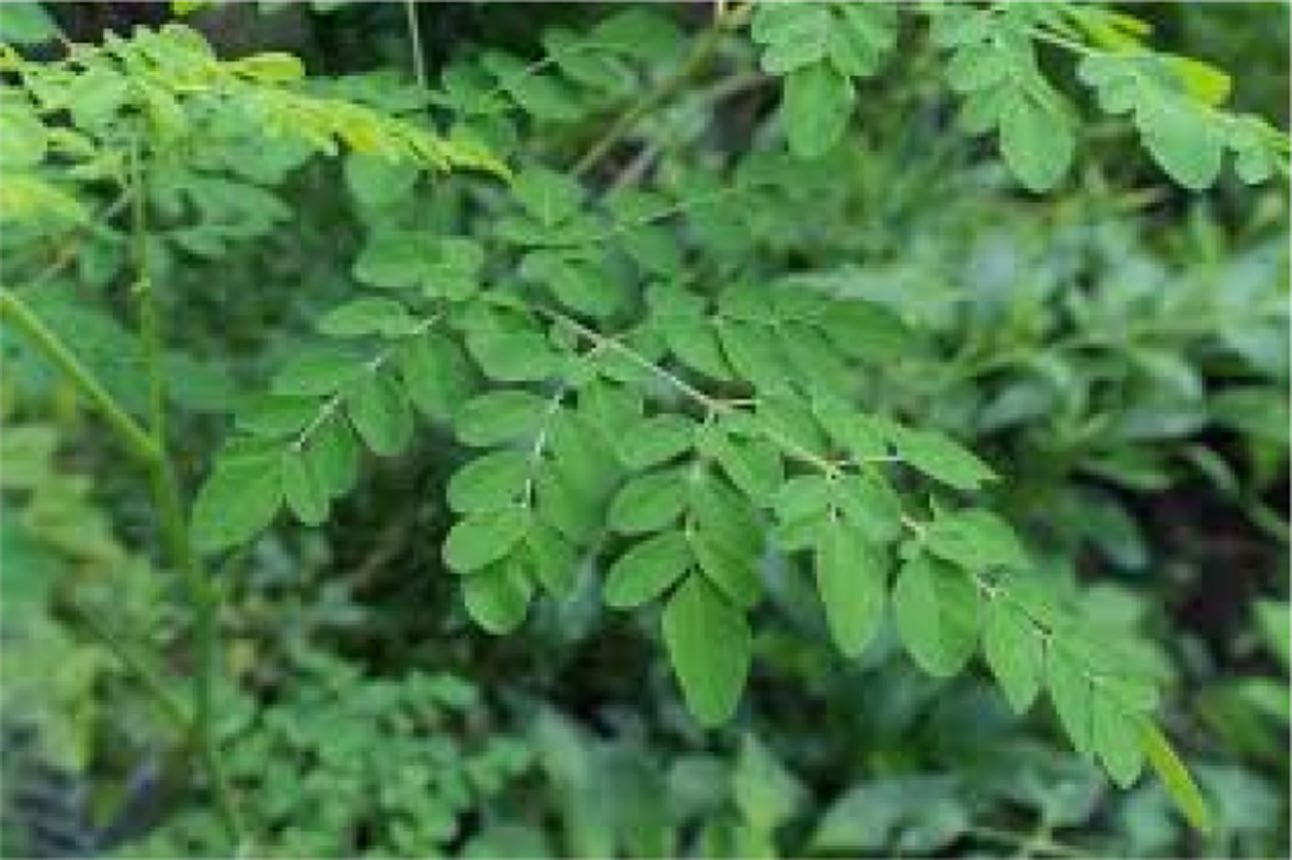

Several studies have demonstrated an extensive use of *M. oleifera* particularly is South Africa is used as a leaf-meal feed supplement to support fertility and semen quality in livestock (30, 31). In a study by Liang et al. (32), in male cashmere goats it was observed that oral *M. oleifera* leaf ethanolic extract (40 mg/kg body weight) or leaf powder (200 mg/kg body weight) improves semen quality (motility, concentration) alongside higher antioxidant capacity, with authors linking effects to shifts in rumen microbiota and metabolites. Early-life supplementation with *M. oleifera* polysaccharides in goat kids has also improved growth and immune indices, suggesting a plausible indirect pathway toward better later reproductive performance via health and development (54) Beyond primary studies, recent literature has provided evidence that *M. oleifera’*s dense nutrient profile (protein, vitamins, minerals) and antioxidant phytochemicals has the potential to improve small-ruminant reproductive indices and semen traits, while also supporting postpartum recovery (55). Complementary evidence from ovine work shows that adding *M. oleifera* extracts to semen extenders improves post-thaw sperm motility and membrane integrity, reinforcing the plant’s antioxidant mechanism-of-action relevant to male gamete function (56) Taken together, although *M. oleifera* is not indigenous to South Africa, its wide local availability, established feed use, and mechanistic evidence make it a practical candidate for goat reproduction programs.

#### 3.3.3 *Elephantorrhiza elephantina* (Burch.) Skeels (elephant root)

**Figure:**
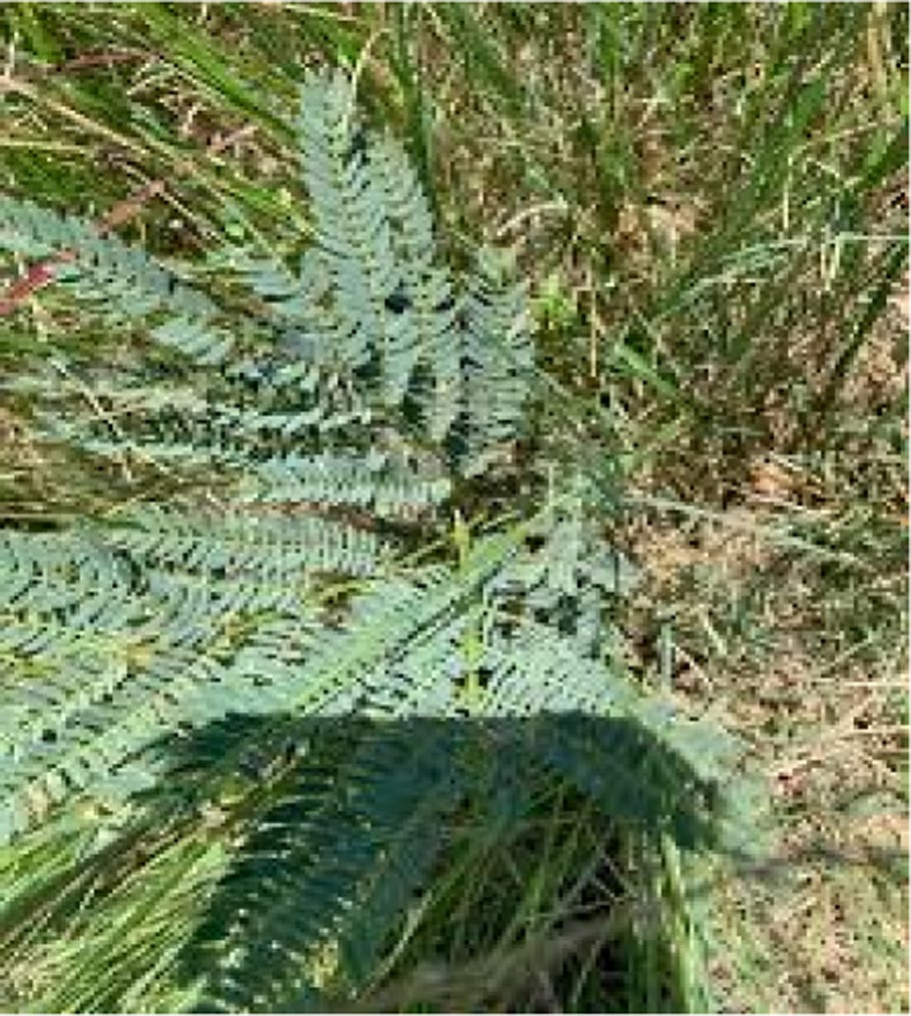

*E. elephantina* is popularly used among the communal farmers in South Africa, particularly in the Eastern Cape and Limpopo provinces, as an anthelmintic in goats. Decoctions of the root are commonly administered to control gastrointestinal parasites, which are among the most important constraints to smallholder goat production. Ethnoveterinary surveys consistently report its use for helminth control, and recent experimental studies provide *in vitro* and *in vivo* validation of its activity against gastrointestinal nematodes such as *Haemonchus contortus* (19, 33). By reducing parasite burdens, *E. elephantina* indirectly supports reproductive performance: parasite control improves body condition, restores nutrient partitioning to reproductive processes, and thereby enhances cyclicity in does and libido in bucks. While the evidence is promising, systematic goat-specific trials are still needed to establish safe dosage, preparation protocols, and long-term reproductive benefits.

#### 3.3.4 . *Kigelia africana* (lam.) Benth.

**Figure:**
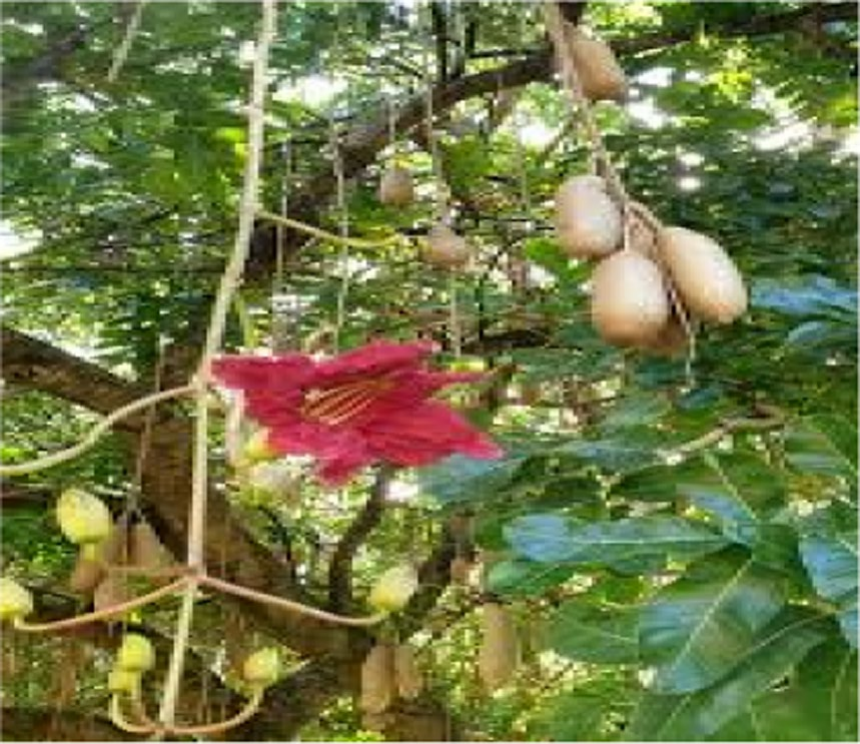

*K. africana* is used in most parts of Africa, particularly in South Africa it is used after parturition for the management and care of the uterus in livestock and is often regarded as “womb cleansing” including management of retained placenta and mastitis (34). Its fruits or bark are prepared as decoction or powders to manage these conditions. Ethnobotanical manuals and compendia explicitly list *K. africana* among ethnoveterinary remedies for retained placenta and mastitis in ruminants, while South African surveys show that retained placenta is a common indication for ethnoveterinary treatment in communal systems (34, 35). Pharmacology reviews report antimicrobial and anti-inflammatory activities that align with these postpartum uses (36).

#### 3.3.5 *Aloe ferox* mill.

**Figure:**
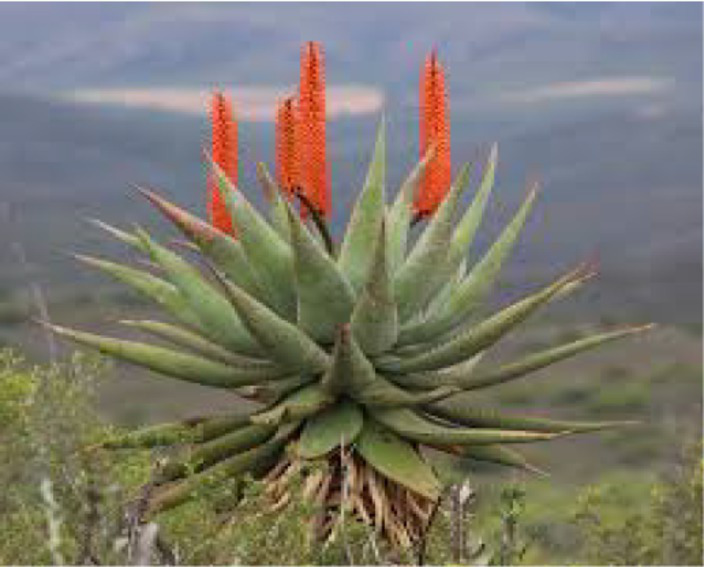

South African Ethnoveterinary practice studies documented the extensive use of *A. ferox*, notably *A. ferox*, as a postpartum uterine care often called the uterine cleansing tonic and for parasite control in small ruminants, with the latter offering indirect fertility benefits via better body condition Comprehensive reviews of *A. fexox* use for animal diseases across Africa document these indications and their prominence in South Africa’s rural livestock systems (37). *In vivo* work in lambs further supports the anthelmintic/production link: supplementation with *A. ferox* leaf material reduced fecal egg counts and increased average daily gain over a 70-day period under gastrointestinal nematode pressure, consistent with improved nutrient partitioning and growth that can precede reproductive gains (38) Ethnobotanical studies in the region also record *A. ferox* among plants used around parturition and for udder/uterine health, aligning with its traditional postpartum applications (39).

## 4 Mechanisms of action

The beneficial effects of South African ethnoveterinary plants on goat reproductive performance can be explained through several overlapping biological mechanisms, ranging from direct impacts on gametogenesis and hormonal regulation to indirect improvements via parasite control and nutrition.

### 4.1 Antioxidant and anti-inflammatory mechanisms

Several studies have demonstrated the extreme susceptibility of sperm cells and testicular tissues to oxidative stress because of the high number of polyunsaturated fatty acids in sperm membranes. Excessive reactive oxygen species (ROS) damage sperm DNA, reduce motility, and impair acrosome reactions (57). Plants such as *M. oleifera*, *S. longepedunculata*, and *A. ferox* contain high levels of polyphenols, flavonoids, and xanthones that scavenge ROS, improve total antioxidant capacity, and stabilize sperm plasma membranes (13, 32, 37). These plants have the capability to protect the testes and uterus from inflammation, due to their Anti-inflammatory compounds, thus, protecting Sertoli and Leydig cell function, which is essential for testosterone production and spermatogenesis (28).

### 4.2 Endocrine modulation

Phytochemicals such as saponins, sterols, and isothiocyanates in *M. oleifera* and *S. longepedunculata* have been shown to influence the hypothalamic pituitary gonadal axis. Several ruminant studies reported possible reproductive enhancement with *M. oleifera* supplementation, noted by increased circulating testosterone, luteinising hormone, and follicle stimulating hormone, which support spermatogenesis, libido, and follicular growth (12, 18). Similarly, phytoestrogens and plant-derived sterols can bind to hormone receptors, mimicking or modulating endogenous hormonal activity (60). These effects may explain traditional reports of enhanced libido and improved estrus expression following plant supplementation.

### 4.3 Antimicrobial and uterotonic actions

Reproductive inefficiency in goats is often linked to uterine infections, retained placenta, and postpartum disorders. Plants such as *K. africana* and *A ferox*. Contain iridoids, anthraquinones, and phenolic compounds with broad spectrum antimicrobial activity against bacteria commonly implicated in metritis and mastitis (35). These compounds may reduce bacterial load in the reproductive tract, supporting uterine involution. In addition, certain phytochemicals act as uterotonics, stimulating uterine contractions that help expel retained placenta and restore normal reproductive function (49). This postpartum effect reduces the “open period” between parturition and subsequent conception.

### 4.4 Anthelmintic and general health pathways

Endoparasites, particularly *Haemonchus contortus*, are a major constraint to goat fertility in South Africa, causing anemia, reduced body condition, and delayed cyclicity. *Elephantorrhiza elephantina* roots are rich in tannins and proanthocyanidins, which bind to parasite cuticles and digestive enzymes, impairing their survival (33). *A. ferox* also exhibits anthelmintic effects, reducing fecal egg counts in lambs and improving average daily gain under parasite challenge (37). By lowering parasite burdens, these plants indirectly support reproductive success through improved nutrient absorption, restored body condition, and enhanced resilience of does during gestation and lactation.

### 4.5 Nutritional support

Literature denotes that there is a direct association between nutrition and reproductive health in livestock (40, 41). *M. oleifera* leaves are a valuable source of crude protein (20–30%), essential amino acids, vitamins (A, C, E), and minerals (Calcium, Iron, Zinc, Selenium). These nutrients are critical for gametogenesis, embryo survival, and postpartum recovery (42). For instance, zinc and selenium act as cofactors in antioxidant enzymes such as glutathione peroxidase, while vitamin A supports follicular development and embryonic survival. Thus, incorporating *M. oleifera* into goat diets provides both pharmacological and nutritional support for reproduction.

## 5 A summary of South African traditional plants used for goat reproductive health

The review identified a range of South African medicinal plants traditionally used to improve reproductive performance in goats. Among the most frequently cited were *S. longepedunculata. M. oleifera*, *E. elephantina, K. africana*, and *A. ferox*. The mechanisms of action reported included antioxidant, endocrine, antimicrobial, anthelmintic, and nutritional pathways. Importantly, very few studies directly examined reproductive outcomes in South African goats, and most findings were extrapolated from indirect health improvements (Table 2).

**Table 2:** South African traditional plants used for goat reproductive health.

Plant name	Part used	Preparation method	Indications	Mechanistic note	References
*Securidaca longepedunculata* Fresen	Root and Leaf	Decoction/maceration	Aphrodisiac/libido	Antioxidant; putative endocrine effects	(13, 20)
*Moringa oleifera* Lam	Leaf	Aqueous extract; Meal ethanolic	Semen quality; libido; post-partum recovery due to nutritional effects	Antioxidant; endocrine modulation; nutrient- dense	(32)
*Elephantorrhiza elephantina*	Root	Decoction	Anthelmentic, indirect fertility via body condition	Tannin rich: parasite suppression supports fertility	(44)
*Kigelia africana*	Fruit/Bark	Meal/decoction	Retained placenta; uterine mastitis	Antimicrobial; anti-inflammatory; Uterine recovery	(35)
*Gnidia capitata*	Leaf/bark	decoction	Uterine cleansing	Antimicrobial; anti-inflammatory	(45)
*Hypoxis hemerocallidea*	Tuber	Immune tonic	Immune support	Sterols	(15, 58)
*Rhoicissus tomentosa*	Root	Infusion	Fertility; abortion; retained placenta	–	
*Aloe ferox* Mill	Leaf	Exudated gel; decoction	Parasite suppression; Uterine cleansing;	Inflammatory	(37)
*Comphocarpus fruticosus*	Root	–	Retained placenta	Antibacterial	(46)
*Asparagus macowani*	Bark	Decoction	Fertility; induce estrus	–	(58)
*Laportea peduncularis*	Root	Decoction	Twin/triplet production	–	(58)
*Duvernoia adhatodoides*	Leaf	Decoction	Fertility/milk letdown		(58)
*Senna italica mill*	Root	–	Retained placenta	–	(6)
*Boophone distcha*	Bulb	Decoction	Uterine cleansing		(6)
*Cochlospermum planchonii rhizome*	Leaf		Increase sperm concentration, length and diameter of testis	Saponins, steroids, terpenoids	(47)

Where goat-specific reproductive trials in South Africa are not yet available, we classify the plant as ethnobotanical/indirect and point to plausible pathways that justify controlled evaluation.

## 6 An overview of the key insights

This review highlights that South Africa possesses a rich repository of ethnoveterinary plants with potential to improve goat reproductive performance, yet most evidence remains anecdotal or indirect. The repeated mention of *S. longepedunculata*, *M. oleifera*, *E. elephantina*, *K. Africana,* and *A ferox*, across ethnobotanical surveys underscores their cultural and practical importance to communal farmers. These findings are consistent with earlier surveys in the Eastern Cape and Limpopo, which reported that over 70% of smallholder farmers rely on medicinal plants to manage reproductive and health challenges in goats (19).

The outstanding outcome of this review is the two-fold role of the indigenous plants, where they demonstrate plausible direct and indirect pathways. The direct reproductive enrichment is associated with the antioxidant and endocrine-modulating effects, as demonstrated with *M. oleifera* and *S. longepedunculata* (13, 32). These phytochemicals reduce oxidative stress, protect sperm membranes, and stimulate reproductive hormones, thereby supporting spermatogenesis and libido (57). Similarly, uterotonic and antimicrobial properties of *K. africana* and *A. ferox* provide plausible explanations for their reported efficacy in managing retained placenta and postpartum infections (35, 37). Indirect pathways, such as parasite suppression by *E. elephantina* and *A. ferox*, improve body condition and nutrient partitioning, which are essential precursors for successful conception and sustained fertility (33, 38).

Despite these promising mechanisms, the review confirms that direct goat-specific studies in South Africa are scarce. Much of the evidence is extrapolated from ethnobotanical accounts or studies conducted in other African regions and species (15). For example, although *M. oleifera* has been shown to improve semen traits and antioxidant status in cashmere goats and rams (32, 43), controlled South African trials measuring libido, semen kinematics, conception rate, and kidding performance are still missing. Similarly, the pharmacological validation of *S. longepedunculata* demonstrates strong antioxidant and antimicrobial activity (13), but toxicity concerns particularly with root extracts pose significant risks (29).

Another key gap revealed by the results is the lack of standardized preparation and dosage protocols. Remedies are typically administered as decoctions, infusions, or powders, with wide variation in concentrations and frequency of use (11). This not only reduces reproducibility but also increases the risk of toxicity, as highlighted for *S. longepedunculata* (20). The absence of phytochemical fingerprinting and dose response trials further hinders regulatory acceptance and extension into mainstream veterinary practice (37).

The results also align with broader discussions on the value of integrating ethnoveterinary medicine with conventional management. For instance, parasite control using *E. elephantina* or *A. ferox*. is most effective when combined with mineral supplementation and improved buck selection, rather than being used in isolation (33). This integrated approach is essential given the multifactorial nature of reproductive inefficiency, where nutrition, parasitism, environment, and management interact (25).

## 7 Conclusion

This review highlights that several South African medicinal plants particularly *S. longepedunculata*, *M. oleifera*, *E. elephantina*, *K. africana*, and *A. ferox.* Hold promise for improving goat reproductive performance through antioxidant, antimicrobial, endocrine-modulating, and anthelmintic effects. However, evidence remains largely ethnobotanical and indirect, with few controlled goat-specific studies. The main limitations of our study include the scarcity of standardized dosing data, limited toxicological information, and inconsistent preparation methods. These can be overcome through well-designed *in vivo* studies, phytochemical profiling, and community-based participatory research to align indigenous knowledge with scientific validation. The review faced difficulties due to fragmented data and scarce goat-focused literature, which were addressed by using multiple research databases and cross-referencing related studies. Future work should prioritize experimental trials on key plants, standardization of safe dosages, and the creation of an ethnoveterinary knowledge repository to preserve and validate traditional practices.
